# AMPK facilitates the hypoxic ventilatory response through non-adrenergic mechanisms at the brainstem

**DOI:** 10.1007/s00424-022-02713-8

**Published:** 2022-06-10

**Authors:** Sandy MacMillan, A. Mark Evans

**Affiliations:** Centre for Discovery Brain Sciences, College of Medicine and Veterinary Medicine, Hugh Robson Building, University of Edinburgh, Edinburgh, EH8 9XD UK

**Keywords:** AMPK, Hypoxic ventilatory response, Apnoea, Hypoxia, Adrenergic, Catecholaminergic

## Abstract

We recently demonstrated that the hypoxic ventilatory response (HVR) is facilitated by the AMP-activated protein kinase (AMPK) in catecholaminergic neural networks that likely lie downstream of the carotid bodies within the caudal brainstem. Here, we further subcategorise the neurons involved, by cross-comparison of mice in which the genes encoding the AMPK-α1 (*Prkaa1*) and AMPK-α2 (*Prkaa2*) catalytic subunits were deleted in catecholaminergic (TH-Cre) or adrenergic (PNMT-Cre) neurons. As expected, the HVR was markedly attenuated in mice with AMPK-α1/α2 deletion in catecholaminergic neurons, but surprisingly was modestly augmented in mice with AMPK-α1/α2 deletion in adrenergic neurons when compared against a variety of controls (TH-Cre, PNMT-Cre, AMPK-α1/α2 floxed). Moreover, AMPK-α1/α2 deletion in catecholaminergic neurons precipitated marked hypoventilation and apnoea during poikilocapnic hypoxia, relative to controls, while mice with AMPK-α1/α2 deletion in adrenergic neurons entered relative hyperventilation with reduced apnoea frequency and duration. We conclude, therefore, that AMPK-dependent modulation of non-adrenergic networks may facilitate increases in ventilatory drive that shape the classical HVR, whereas AMPK-dependent modulation of adrenergic networks may provide some form of negative feedback or inhibitory input to moderate HVR, which could, for example, protect against hyperventilation-induced hypocapnia and respiratory alkalosis.

**Supplementary Information:**

The online version contains supplementary material available at 10.1007/s00424-022-02713-8.

## Introduction

We recently identified a role for the AMP-activated protein kinase (AMPK) in regulating breathing and thus oxygen supply [11, 37] by facilitating the hypoxic ventilatory response (HVR) [38]. Classically, AMPK has been regarded as a cellular energy sensor that acts to maintain energy homeostasis in a cell-autonomous manner. Cells may express as many as twelve AMPK isoforms through the heterotrimeric associations of one from each of the 2 α-catalytic and 2 β- and 3 γ-regulatory subunits. AMPK is coupled to mitochondrial oxidative phosphorylation, and thus oxygen supply, by two discrete albeit cooperative pathways. Binding of AMP to the AMPK γ-subunit increases activity tenfold by allosteric action, while AMP or ADP binding delivers increases in LKB1-dependent phosphorylation and reductions in dephosphorylation of Thr172 on the α-subunit that confer 100-fold further activation. All of these effects are inhibited by ATP [13]. There is also an alternative calcium-dependent pathway to AMPK activation that is governed by the calmodulin-dependent protein kinase CaMKK2, which delivers increases in Thr172 phosphorylation and thus AMPK activation independent of changes in cellular AM(D)P:ATP ratios. Importantly, each AMPK isoform may hold different sensitivities to activation by increases in cellular AMP and ADP and differ in their capacity to directly phosphorylate and thus regulate downstream targets [27].

Our studies suggest that the role of AMPK is not limited to cellular metabolic homeostasis but extends to breathing and thus oxygen [34, 36] and energy (ATP) supply to the body as a whole. However, in this respect, AMPK does not appear to act at the level of the carotid bodies as one would predict given their role as the primary arterial chemoreceptors [14, 25, 29, 34], but downstream and most likely at the caudal brainstem [23]. Located within the caudal portions of the brainstem are the noradrenergic A1 and A2 cell groups, which receive peripheral carotid body afferent inputs and relay these signals to the respiratory central pattern generators (rCPG) [17, 30, 34]. The rostral portion of the brainstem on the other hand contains the adrenergic (phenylethanolamine N-methyltransferase (PNMT) expressing) C1, C2, and C3 cell groups, which also contribute to sympathoexcitatory responses and the regulation of breathing [15]. The dorsal C2 and C3 groups are located bilaterally and medially, respectively, in rostral regions of the NTS, while the C1 group is located bilaterally in the ventral medulla where the majority of brainstem PNMT-positive neurons are located [24]. Of these, it is evident that C1 neurons innervate, among other sites, the respiratory centres of the brainstem, sympathetic preganglionic neurons in the spinal cord, and the dorsal motor nucleus of the vagus (10 N) [16]. Furthermore, optogenetic stimulation of C1 cells has been shown to increase breathing frequencies in conscious mice [2] and rats [5], mostly through direct projections to the retrotrapezoid nucleus (RTN), which in turn modulates respiratory output through the rCPG [1, 5, 6].

Our previous investigations into the role of AMPK in facilitating the HVR relied upon a gene deletion strategy that targeted all catecholaminergic neurons through Cre recombinase expression via the tyrosine hydroxylase promoter [23, 38]. To gain further insight, the present study compares outcomes for these mice with those for mice in which AMPK deletion was targeted to adrenergic neurons through Cre expression via the gene that encodes PNMT (*Pnmt*). We show here that while AMPK-α1/α2 deletion in catecholaminergic neurons precipitated marked hypoventilation and apnoea during poikilocapnic hypoxia, AMPK deletion in adrenergic neurons modestly augments the HVR and reduces apnoea frequency. In short, AMPK facilitates the HVR through non-adrenergic mechanisms.

## Methods

### Mouse models

Experiments were performed in accordance with the regulations of the United Kingdom Animals (Scientific Procedures) Act of 1986. All studies and breeding were approved by the University of Edinburgh and performed under UK Home Office project licenses. For the main study described here, both male and female mice were used. All were on a C57/Bl6 background, with the exception of a secondary control experiment on mice developed on a CD1 background (Supplementary Figs. 2–5). Numbers of mice (≥ 3 per measure) used are indicated for each experiment. Global, dual knockout of the genes encoding AMPK-α1 (*Prkaa1*) and AMPK-α2 (*Prkaa2*) is embryonic lethal. We therefore employed conditional deletion of the genes for the AMPK-α1 and AMPK-α2 subunits, using mice in which the sequence encoding the catalytic site of both α-subunits was flanked by loxP sequences [21]. To direct AMPK deletion to adrenergic cells, we crossed these ‘floxed’ mice with a transgenic mouse model in which the sequence encoding Cre recombinase was inserted into the 5′ UTR of the phenylethanolamine N-methyltransferase (*Pnmt*) gene (a kind gift from Prof Steven Ebert, University of Central Florida) [10]. Mice with AMPK deletion in catecholaminergic cells were obtained as described previously [38] by crossing floxed mice with mice in which Cre recombinase expression was driven via the tyrosine hydroxylase (TH) promoter [22].

### Genotyping

The presence of wild-type or floxed AMPK-α1 and AMPK-α2 alleles and Cre recombinase (Supplementary Fig. 1) was detected by PCR. We used three primers for TH Cre (forward 5′ CACCCTGACCCAAGCACT 3′; reverse 5′ CTTTCCTTCCTTTATTGAGAT 3′; internal positive control 5′ GATACCTGGCCTGGTCTCG 3′; expected size WT = 290 bp, Cre = 390 bp) and PNMT Cre (21, 5′ CAGGCGCCTCATCCCTCAGCAGCC 3′; 22, 5′ CTGGCCAGCGTCGGAGTCAGGGTC 3’; and 23, 5′ GGTGTACGGTCAGTAAATTGGACACCGTCCTC 3′; expected size WT = 190 bp, Cre = 300 bp). Two primers were used for each AMPK catalytic subunit: α1-forward 5′ TATTGCTGCCATTAGGCTAC 3′, α1-reverse 5′ GACCTGACAGAATAGGATATGCCCAACCTC 3′ (WT = 588 bp, floxed = 682 bp) and α2-forward 5′ GCTTAGCACGTTACCCTGGATGG 3′, α2-reverse 5′ GTTATCAGCCCAACTAATTACAC 3′ (WT = 204 bp, floxed = 250 bp). Ten microlitres of samples was run on 2% agarose gels with 0.01% v/v SYBR®Safe DNA Gel Stain (Invitrogen) in TBE buffer against a 100 bp DNA ladder (GeneRuler™, Fermentas) using a Model 200/2.0 Power Supply (Bio-Rad). Gels were imaged using a Genius Bio Imaging System and GeneSnap software (Syngene).

### Plethysmography

As described previously [34, 36], we used a whole-body unrestrained plethysmograph, incorporating a Halcyon™ low-noise pneumotachograph (Buxco Research Systems, UK) coupled to FinePointe acquisition and analysis software (Data Science International, USA). Following acclimation and baseline measurements (awake but quiet, undisturbed periods of breathing) under normoxia (room air), mice were exposed to hypoxia (8% O_2_, with 0.05% CO_2_, balanced with N_2_) for 10 min. The FinePointe software automatically calculated the respiratory parameters assessed after application of exclusion criteria due to non-ventilatory artefacts (movement, sniffing, etc.). Data were acquired as 2-s averages, and 2 to 4 data points of undisturbed breathing were selected for each time point of the HVR. Apnoeas were defined as a period of cessation of breathing that was greater than the average duration, including inter-breath interval, of 2 successive breaths (~ 600 ms) of control mice during normoxia with a detection threshold of 0.25 mmHg (SD of noise).

### Confocal imaging

To identify neuronal networks in which dual AMPK-α1/α2 deletion had been induced, mice with PNMT-Cre-driven AMPK-α1/α2 deletion and TH-Cre-driven AMPK-α1/α2 deletion were crossed with mice engineered for Cre-dependent expression of Rosa (tdTomato). These mice were deeply anaesthetised using 2 g/kg Pentobarbital Sodium (Merial), transcardially perfused with ice-cold heparinised saline and fixed with 4% paraformaldehyde in 0.1 M phosphate buffer (PB; pH 7.4). Brains were extracted, post-fixed, and stored in 30% sucrose in 0.1 M PB at 4 °C. Thirty-micrometre sections of the brainstem were cut using a Frigomobil freezing microtome (Leica). Alternate sections were collected together and mounted onto glass slides, briefly air-dried, and coverslipped using Vision™ PermaFluor™ Aqueous Mounting Medium (Thermo Fisher).

Brainstem sections were imaged using a Nikon A1R + confocal system and tdTomato autofluorescence detected using an excitation wavelength of 554 nm and emission wavelength of 581 nm. Relevant regions of the caudal brainstem harbouring catecholaminergic neurons were identified using the mouse brain atlas [39].

### Statistical analysis

Statistical comparison was completed using GraphPad Prism 6. For plethysmography, one-way ANOVA with Tukey post hoc test was used when comparing across one variable (e.g. genotype) and two-way ANOVA with Sidak post hoc tests when comparing across two variables (e.g. genotype and time). Statistical significance was assumed when *p* ≤ 0.05.

## Results

The original cohort of PNMT-Cre mice we received was bred on a CD1 background, whereas the TH-Cre and AMPK-α1/α2 floxed mice were bred on a C57Bl6 background. Therefore, the CD1 PNMT-Cre mice were backcrossed for at least four generations onto a C57Bl6 background, to eliminate genetic differences between background strains that influence the hypoxic ventilatory response (HVR) (Supplementary Figs. 2–5; see also [3, 19, 32, 33, 35]). We then selected for AMPK-α1/α2 deletion in adrenergic cells [21] by heterozygous PNMT-Cre expression, which is sufficient for cell-specific excision of AMPK-α1/α2 [38], and thus avoided loss of PNMT expression and consequent adrenaline deficiency that is known to be associated with homozygous insertion of Cre at exon 1 of *Pnmt* [10].

**Fig. 1:**
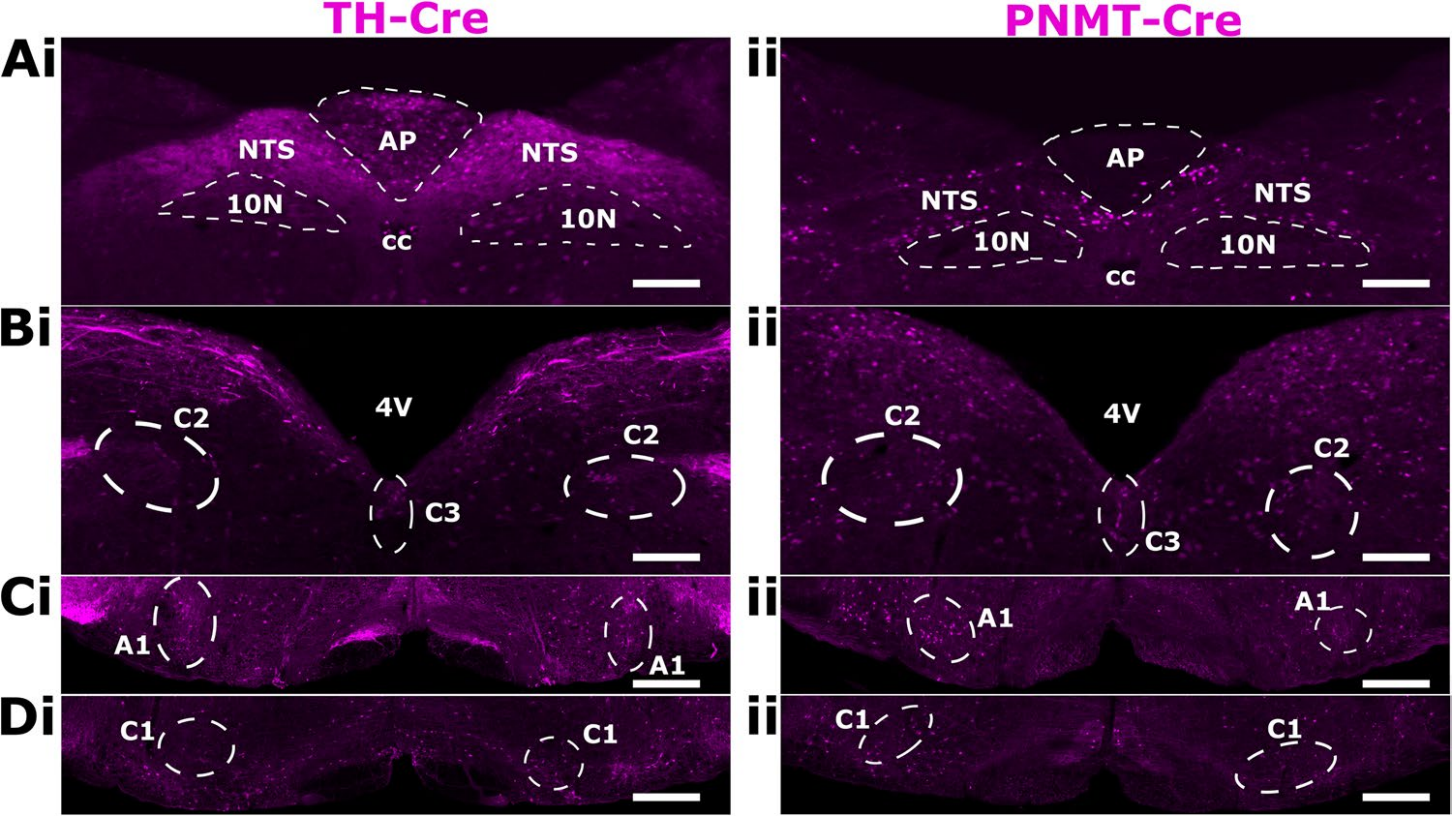
Representative confocal images of Cre-driven Rosa fluorescence in catecholaminergic and adrenergic neurons of AMPK-α1/α2 knockout mice. **a** Exemplar images of brainstem sections through the dorsal nucleus tractus solitarius (NTS) at the level of the area postrema (AP), central canal (cc), and the dorsal motor nucleus of the vagus (10 N; approximate Bregma − 7.5 mm) from a mouse in which AMPK-α1/α2 deletion and tdTomato expression (magenta) was targeted to (i) catecholaminergic cells by Cre expression via the tyrosine hydroxylase promoter (TH-Cre) and (ii) adrenergic cells by Cre expression via the phenylethanolamine N-methyltransferase promoter (PNMT-Cre). **b** As for (a) but showing the C2 and C3 neuronal groups at the level of the 4th ventricle (4 V; approximate Bregma − 6.7 mm). **c** As for (a) and (b) but showing the A1 neuronal groups of the ventrolateral medulla (approximate Bregma − 7.5 mm). **d** As for (a–c) but showing the C1 neuronal groups of the ventrolateral medulla (approximate Bregma − 6.7 mm). Scale bars are 200 µm

Cell-specific gene deletion was confirmed previously for TH-positive cells by single-cell endpoint reverse transcription-polymerase chain reaction and whole-brain quantitative polymerase chain reaction, and restriction of Cre to TH-positive cells in the adult mouse was verified by viral transfection of a Cre-inducible vector carrying a reporter gene [38]. Here, we additionally crossed TH-AMPK-α1/α2 knockouts and PNMT-AMPK-α1/α2 knockouts with mice expressing the Cre-inducible reporter gene Rosa (tdTomato), the expression of which was assessed by confocal imaging to identify cells. In accordance with the discrete distribution of adrenergic C1, C2, and C3 neurons within the brainstem when compared to nor-adrenergic A1 and A2 neurons [15, 17, 20], qualitative comparison of thirty-micrometre brain sections revealed (Fig. 1) marked regional variations in Rosa expression between PNMT-AMPK-α1/α2 knockouts and TH-AMPK-α1/α2 knockouts at, for example, the area postrema (AP) and across proximal regions of the dorsal nucleus tractus solitarius (NTS), as one would expect given the distribution of adrenergic and noradrenergic neurons at the brainstem [20].

### AMPK deletion in catecholaminergic but not adrenergic cells attenuates the hypoxic ventilatory response

**Fig. 2:**
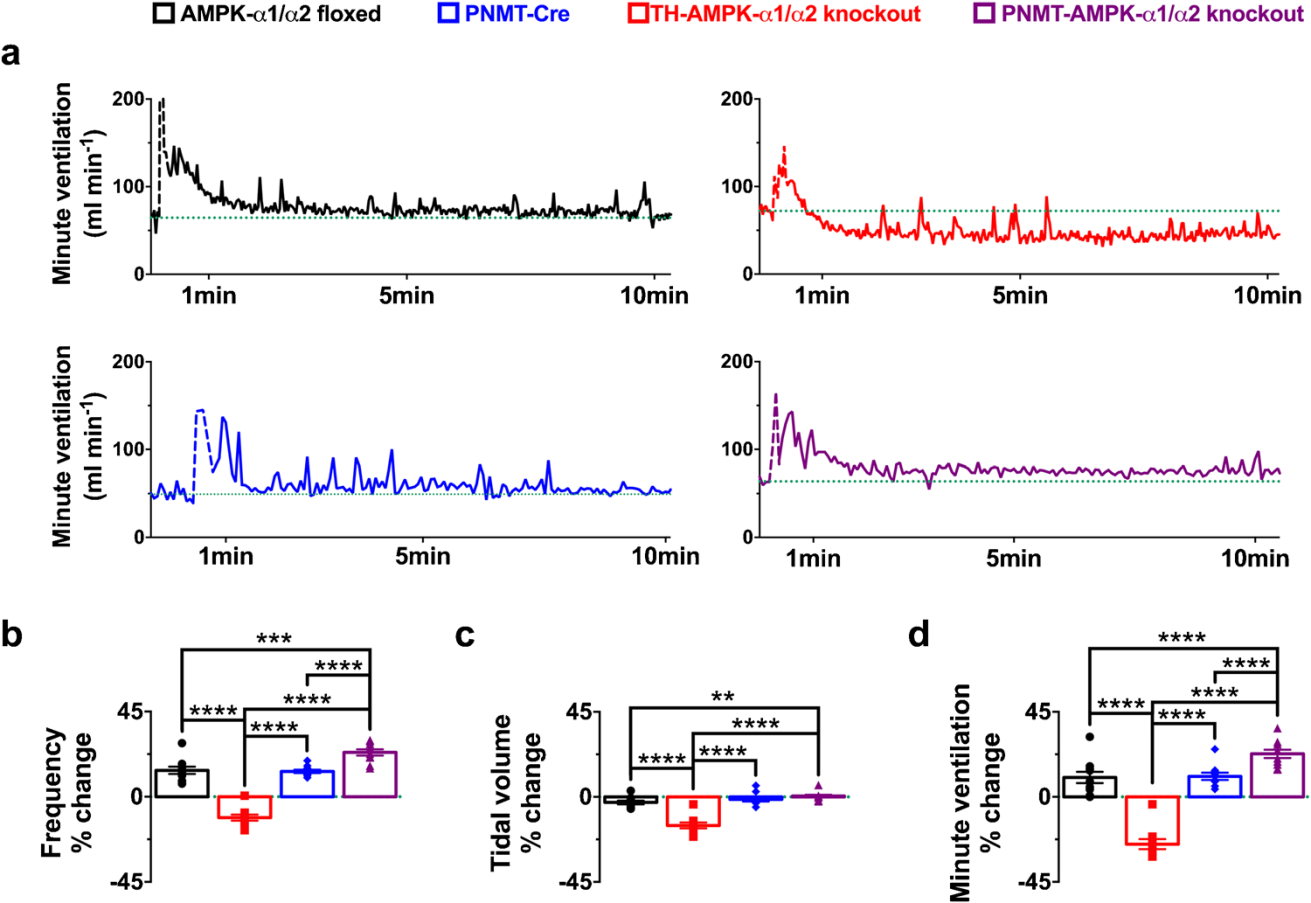
AMPK deletion in adrenergic cells augments and AMPK deletion in catecholaminergic cells attenuates the hypoxic ventilatory response. **a** Example records of minute ventilation (ml min.^−1^) versus time during exposures to severe hypoxia (8% O_2_) of AMPK-α1/α2 floxed mice (black, *n* = 58 exposures from 21 mice), mice with conditional deletion of AMPK-α1/α2 in catecholaminergic cells (TH-AMPK-α1/α2 knockout, red, *n* = 49 exposures from 21 mice), mice expressing Cre under the phenylethanolamine N-methyltransferase reporter (PNMT-Cre, blue, *n* = 32 exposures from 8 mice), and mice with conditional deletion of AMPK-α1/α2 in adrenergic cells (PNMT-AMPK-α1/α2 knockout, purple, *n* = 32 exposures from 8 mice). Bar charts show mean ± SEM for percentage changes relative to normoxia in **b** breathing frequency, **c** tidal volume, and **d** minute ventilation. ** = *p* < 0.01, *** = *p* < 0.001, **** = *p* < 0.0001

**Fig. 3:**
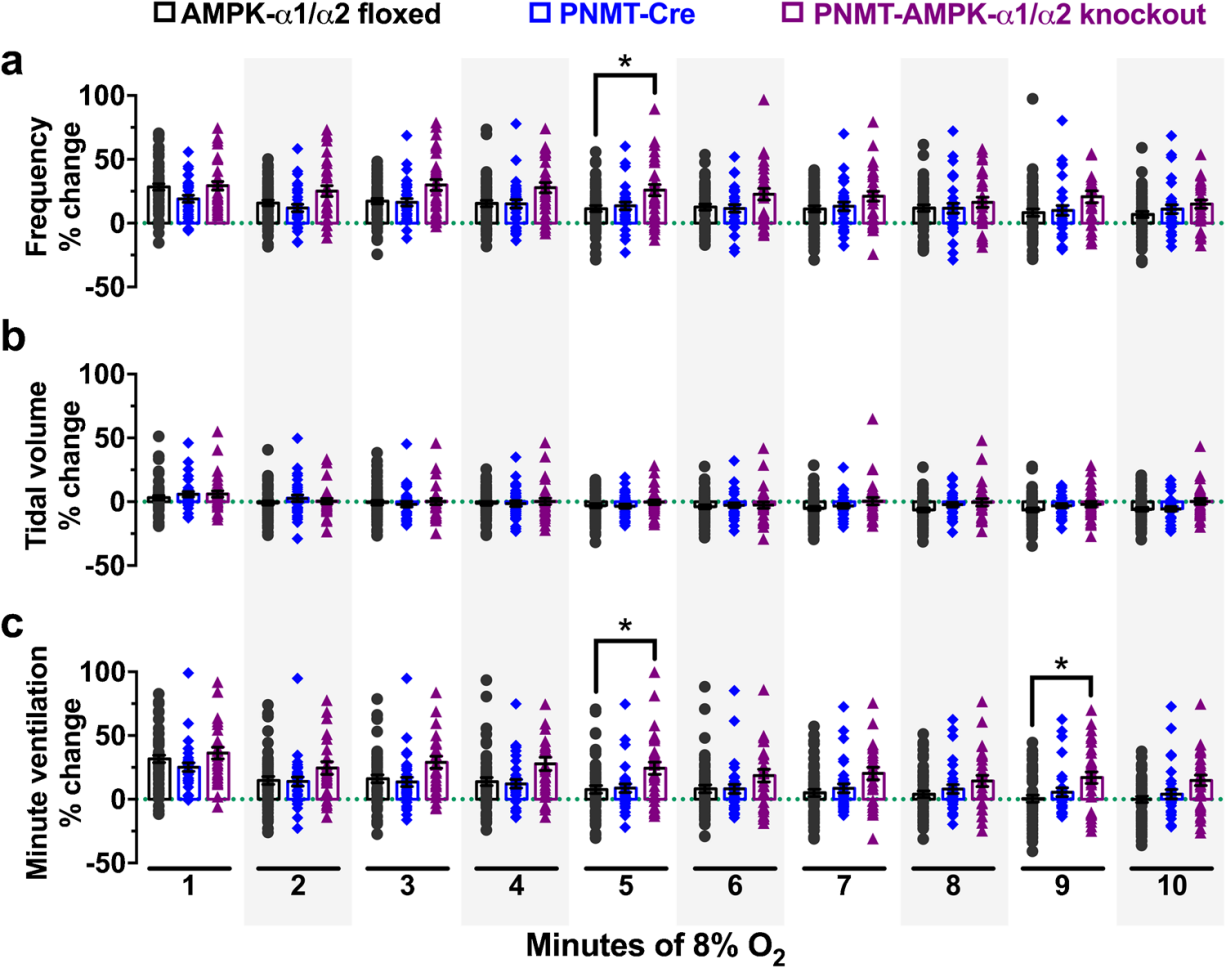
The effect of AMPK deletion in adrenergic cells on the hypoxic ventilatory response to severe hypoxia. Means ± SEM for percentage changes relative to normoxia at each full minute of exposure to severe hypoxia (8% O_2_) of **a** breathing frequency, **b** tidal volume, and **c** minute ventilation during 10-min exposures to severe hypoxia (8% O_2_) in AMPK-α1/α2 floxed mice (black, *n* = 58 exposures from 21 mice), mice expressing Cre under the phenylethanolamine N-methyltransferase reporter (PNMT-Cre, blue, *n* = 32 exposures from 8 mice), and PNMT Cre-driven AMPK-α1/α2 double knockout mice (PNMT-AMPK-α1/α2 knockout, purple, *n* = 32 exposures from 8 mice). * = *p* < 0.05

As reported previously [38] in TH-AMPK-α1/α2 knockouts, the HVR was markedly attenuated relative to AMPK-α1/α2 floxed mice (Fig. 2), which is highlighted here by new exemplar records of minute ventilation (Fig. 2a) and data (mean ± SEM) for breathing frequency, tidal volume, and minute ventilation measured during 10-min exposures to hypoxia (Fig. 2b–d). Analysis of the ventilatory patterns of PNMT-AMPK-α1/α2 knockouts under normoxia revealed no physiologically relevant differences relative to AMPK-α1/α2 floxed mice or backcrossed heterozygous PNMT-Cre mice (Supplementary Fig. 6). In stark contrast, the HVR was moderately augmented in PNMT-AMPK-α1/α2 knockouts when compared to controls during 10-min exposures to severe hypoxia (8% O_2_), and yet more so relative to TH-AMPK-α1/α2 knockouts. Briefly, changes in hypoxic ventilatory parameters relative to normoxia for control AMPK-α1/α2 floxed mice (*n* = 58 exposures from 21 mice) measured 14 ± 1.9% for breathing frequency, − 2.9 ± 1% for tidal volume and 10.3 ± 3% for minute ventilation. In accordance with our previous study [38], all of these parameters were significantly attenuated in TH-AMPK-α1/α2 knockouts (*n* = 49 exposures from 21 mice) relative to controls, with breathing frequency measuring – 11 ± 1.6% (*p* < 0.001), tidal volume − 15.1 ± 1.5% (*p* < 0.001), and minute ventilation – 25 ± 2.6% (*p* < 0.001). By contrast, all ventilatory parameters were significantly augmented in PNMT-AMPK-α1/α2 knockouts (*n* = 32 exposures from 8 mice), where breathing frequency measured 23.4 ± 1.6% (*p* < 0.001), tidal volume 0.4 ± 0.7%, (*p* < 0.01), and minute ventilation 22.8 ± 2.2% (*p* < 0.001). Importantly, this modest augmentation of the HVR in PNMT-AMPK-α1/α2 knockout mice held true whether one used PNMT-Cre (*n* = 32 exposures from 8 mice; breathing frequency 13.4 ± 0.9%, tidal volume − 1.3 ± 1%, minute ventilation 10.9 ± 1.9%) as controls or AMPK-α1/α2 floxed mice. However, although this trend was maintained when data were analysed at 1-min intervals, increases in the HVR only reached significance at few time points when broken down in this way (Fig. 3).

**Fig. 4:**
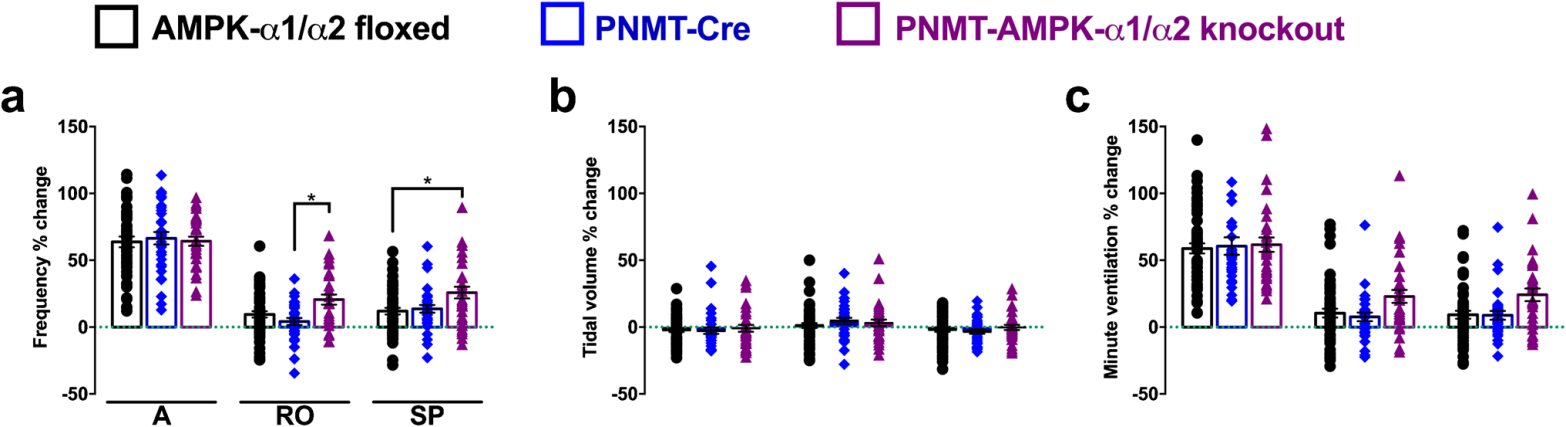
AMPK-α1/α2 catalytic subunit deletion in adrenergic cells augments the hypoxic ventilatory response to severe hypoxia through increases in breathing frequency. Means ± SEM of percentage changes relative to normoxia of **a** breathing frequency, **b** tidal volume, and **c** minute ventilation during exposures to severe hypoxia (8% O_2_) for AMPK-α1/α2 floxed mice (black, *n* = 58 exposures from 21 mice), mice expressing Cre under the phenylethanolamine N-methyltransferase reporter (PNMT-Cre, blue, *n* = 32 exposures from 8 mice), and PNMT Cre-driven AMPK-α1/α2 double knockout mice (PNMT-AMPK-α1/α2 knockout, purple, *n* = 32 exposures from 8 mice) measured at the peak of the augmenting phase (A, approximately 30 s), the nadir of the roll-off (RO, approximately 100 s), and the plateau of the sustained phase (SP, at 300 s). * = *p* < 0.05

More detailed analysis revealed that the initial augmenting phase of the HVR remained largely unaffected in PNMT-AMPK-α1/α2 knockouts relative to PNMT-Cre, whether one considers breathing frequency, tidal volume, or minute ventilation (Fig. 4). For example, upon exposure to severe hypoxia (8% O_2_), increases in breathing frequency at the peak of the augmenting phase for PNMT-AMPK-α1/α2 knockouts measured 64.2 ± 3.4% relative to normoxia while that for PNMT-Cre mice measured 66.5 ± 4.6%. As one might expect, this translated into comparable measures of minute ventilation for PNMT-AMPK-α1/α2 knockouts (61.6 ± 5.4%) and PNMT-Cre (60.6 ± 6.6%). By contrast, following ventilatory decline during roll-off, breathing frequencies of PNMT-AMPK-α1/α2 knockouts were significantly increased (20.5 ± 3.8%, *p* < 0.05) when compared to PNMT-Cre mice (4.2 ± 2.6%), although this did not translate into statistically significant increases in minute ventilation (22.9 ± 4.9% for PNMT-AMPK-α1/α2 knockout versus 7.7 ± 3.2% for PNMT-Cre, *p* = 0.17). Thereafter, these ventilatory parameters for PNMT-AMPK-α1/α2 knockouts remained elevated on average throughout the sustained phase of the HVR during 10-min exposures to severe hypoxia, whether we consider breathing frequency (25.8 ± 4.4%, *p* < 0.05) or minute ventilation (24.2 ± 4.7%, *p* = 0.16) when compared to PNMT-Cre (breathing frequency, 13.6 ± 2.9%; minute ventilation, 8.8 ± 3.3%).

When taken together, the aforementioned findings are in agreement with the view that the augmenting phase of the HVR is primarily mediated by carotid body afferent input responses [8, 34, 36], while direct modulation by hypoxia of brainstem respiratory networks aids maintenance of the HVR in the longer term [7, 23, 28, 34, 36].

### AMPK deletion in catecholaminergic but not adrenergic cells precipitates breathing instabilities

**Fig. 5:**
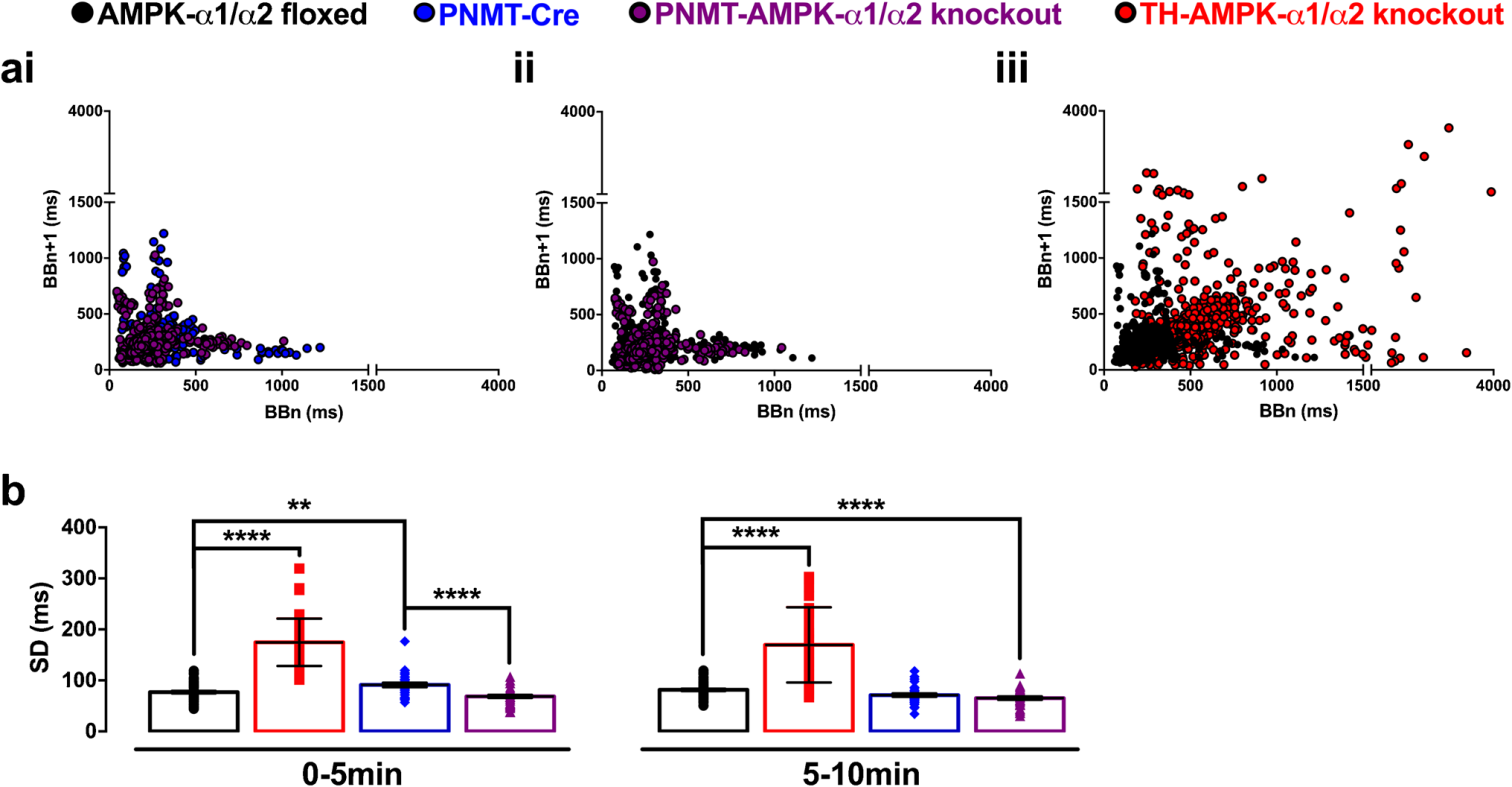
Differences in breathing regularity between mice with deletion of AMPK in adrenergic or catecholaminergic cells. **a** Example Poincaré plots show the inter-breath interval (BBn) versus the subsequent interval (BBn + 1) of individual exposures from (i) mice expressing Cre under the phenylethanolamine N-methyltransferase reporter (PNMT-Cre, blue), (i and ii) PNMT Cre-driven AMPK-α1/α2 double knockout mice (PNMT-AMPK-α1/α2 knockout, purple), (ii and iii) AMPK-α1/α2 floxed mice (black), and (iii) TH-driven AMPK-α1 and -α2 double knockout mice (TH-AMPK-α1/α2 knockout, red) during 10-min exposures to severe hypoxia (8% O_2_). **b** Means ± SEM of the standard deviation (SD) of BBn for AMPK-α1/α2 floxed mice (*n* = 58 exposures from 21 mice), TH-AMPK-α1/α2 knockout mice (*n* = 49 exposures from 21 mice), PNMT-Cre mice (*n* = 32 exposures from 8 mice), and PNMT-AMPK-α1/α2 knockout mice (*n* = 32 exposures from 8 mice) during the first and second 5 min of 10-min exposures to severe hypoxia. ** = *p* < 0.01, **** = *p* < 0.0001

**Fig. 6:**
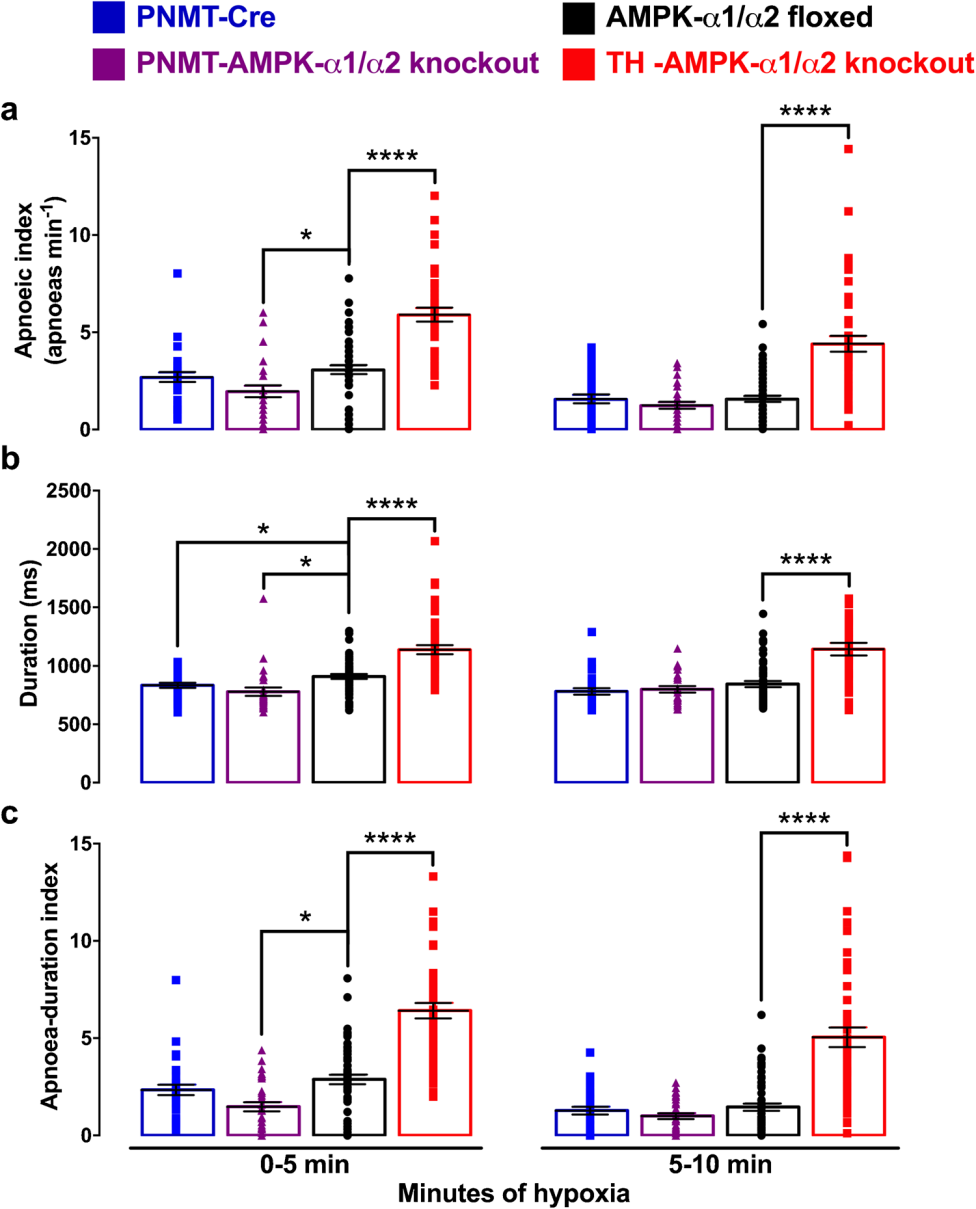
AMPK deletion in adrenergic cells reduces and AMPK deletion in catecholaminergic cells increases apnoeas during exposures to severe hypoxia. Means ± SEM of the **a** apnoeic index (min^−1^), **b** apnoea duration (ms), and **c** apnoea-duration index (frequency x duration) for PNMT-Cre mice (blue, *n* = 32 exposures from 8 mice), PNMT Cre-driven AMPK-α1/α2 double knockout mice (PNMT-AMPK-α1/α2 dKO, purple, *n* = 32 exposures from 8 mice), AMPK-α1/α2 floxed mice (black, *n* = 58 exposures from 21 mice), and TH-Cre-driven AMPK-α1/α2 double knockout mice (TH-AMPK-α1/α2 knockout, red, *n* = 49 exposures from 21 mice). * = *p* < 0.05, **** = *p* < 0.0001

We next assessed the overall breathing regularity and hence stability during hypoxia by Poincaré plots of inter-breath interval (BBn) against each subsequent inter-breath interval (BBn + 1). Example plots from single 10-min exposures are shown in Fig. 5a, which compares the inter-breath intervals of exemplar PNMT-Cre and PNMT-AMPK-α1/α2 knockout mice with those from AMPK-α1/α2 floxed and TH-AMPK-α1/α2 knockout mice exposed to severe hypoxia (8% O_2_). Both the plots and analysis of the standard deviation (SD) of BBn (Fig. 5b) indicated marked breathing instability in TH-AMPK-α1/α2 with increases in apnoea frequency and duration (see also Fig. 6). Briefly, the SD of BBn for TH-AMPK-α1/α2 knockouts was significantly greater than controls within the first (0–5 min: 174.9 ± 6.8 ms, *p* < 0.0001) and second 5-min periods (5–10 min: 169.8 ± 10.9 ms, *p* < 0.0001) of exposure to severe hypoxia when compared to AMPK-α1/α2 floxed mice (0–5 min, 77.2 ± 2.3 ms; 5–10 min, 81.5 ± 2.2 ms). By contrast, breathing stability during hypoxia was enhanced and apnoea less prevalent in PNMT-AMPK-α1/α2 knockouts (0–5 min, 68.7 ± 3 ms; 5–10 min, 65.4 ± 3.3 ms) relative to controls (PNTM-Cre, 0–5 min, 91.3 ± 4.2 ms; 5–10 min, 71.3 ± 3.2 ms), particularly during the first 5 min of each 10-min exposure.

Consistent with this, minute-by-minute analyses revealed significant differences in the SD of BBn and BBn + 1 between TH-AMPK-α1/α2 knockouts when compared to controls or PNMT-AMPK-α1/α2 knockouts. This was particularly so at the beginning of exposures to hypoxia, where the breathing stability of PNMT-AMPK-α1/α2 knockouts was enhanced relative to PNMT-Cre (Supplementary Fig. 8, left panel). Briefly, for PNMT-Cre mice, the SD of BBn measured 69.5 ± 3.4 ms, 104.2 ± 4.4 ms, and 97.5 ± 8.7 ms during the first, second, and third minutes of severe hypoxia, and then stabilised at ~ 70 ms for the remainder of the exposure period. By contrast, for PNMT-AMPK-α1/α2 knockouts, the SD of BBn was initially lower, measuring 52.1 ± 2.6 ms, (*p* < 0.05), 75.8 ± 3.8 ms (*p* < 0.0001), and 67.5 ± 4.3 ms (*p* < 0.0001) during the first, second, and third minutes of 10-min exposures to hypoxia, before stabilising at a slightly lower but not statistically significant level of ~ 60 ms. This is in stark contrast to the impact on inter-breath intervals of deleting AMPK-α1/α2 in all catecholaminergic neurons, where ventilatory instability was markedly increased during hypoxia (Fig. 5aiii–b; see also [38]). Moreover, and consistent with all other genotypes, the highest degree of variability in inter-breath interval was observed during the second minute of severe hypoxia (207.7 ± 13.6 ms, *p* < 0.0001 relative to AMPK-α1/α2 floxed: 108.4 ± 4.4 ms). However, while controls and PNMT-AMPK-α1/α2 knockouts exhibited a time-dependent reduction of the SD for BBn, this was entirely absent in TH-AMPK-α1/α2 knockouts (Supplementary Fig. 8, right panel; AMPK-α1/α2 floxed, 108.4 ± 4.4 ms and TH-AMPK-α1/α2 knockouts, 170.3 ± 15.5 ms at 5–6 min, *p* < 0.0001; AMPK-α1/α2 floxed, 77.9 ± 4.2 ms and TH-AMPK-α1/α2 knockouts, 146 ± 11 ms at 9–10 min, *p* < 0.0001).

### AMPK deletion in adrenergic cells reduces apnoea frequency and duration

As one might expect given the above, apnoea frequency of TH-AMPK-α1/α2 knockouts was markedly increased relative to controls and reduced in PNMT-AMPK-α1/α2 knockouts (Fig. 6a). During the first 5 min of exposures to hypoxia, apnoea frequency measured 1.9 ± 0.3 apnoeas min^−1^ for PNMT-AMPK-α1/α2 knockouts compared to 2.7 ± 0.3 apnoeas min^−1^ for PNMT-Cre (NS) and 3.1 ± 0.2 apnoeas min^−1^ for AMPK-α1/α2 floxed (*p* < 0.05). During the second half of hypoxia, however, only a minor reduction in the apnoea frequency was observed in PNMT-AMPK-α1/α2 knockouts (1.2 ± 0.2 apnoeas min^−1^) that did not reach significance relative to either of the controls (PNMT-Cre, 1.6 ± 0.2 apnoeas min^−1^; AMPK-α1/α2 floxed, 1.6 ± 0.2 apnoeas min^−1^). By contrast, TH-AMPK-α1/α2 knockouts exhibited marked and sustained increases in the apnoeic index relative to AMPK-α1/α2 floxed mice, which measured 5.9 ± 0.4 apnoeas min^−1^ (*p* < 0.0001) during the first half and 4.4 ± 0.4 apnoeas min^−1^ (*p* < 0.0001) during the second half of 10-min exposures.

Apnoea durations for PNMT-AMPK-α1/α2 knockouts (Fig. 6b) during the first (778.7 ± 35.7 ms) and second (799.7 ± 27.5 ms) 5-min periods were comparable to measures for PNMT-Cre mice (834.3 ± 21.8 ms and 781.8 ± 28 ms, respectively), but they were significantly shorter than for AMPK-α1/α2 floxed mice (909.7 ± 21.4 ms, *p* < 0.05) which were in turn significantly longer than for PNMT-Cre mice from 0–5 min (*p* < 0.05). More detailed analysis (Supplementary Fig. 9bi) revealed significant reductions in apnoea duration for PNMT-AMPK-α1/α2 knockouts, but only during the third minute of hypoxia (750.8 ± 24.6 ms, *p* < 0.05) when compared to PNMT-Cre (877.5 ± 27 ms).

In marked contrast, TH-AMPK-α1/α2 knockouts exhibited marked increases in apnoea duration compared to AMPK-α1/α2 floxed mice during the first (1139.4 ± 39.2 ms, *p* < 0.0001) and second (1143.6 ± 54.3 ms, *p* < 0.0001) period of 5-min exposures to hypoxia, consistent with previous observations [38]. Moreover, this increase in apnoea duration was evident at each minute throughout 10-min exposures to hypoxia (1–2 min, 1031 ± 38.9 ms, *p* < 0.0001; at 5–6 min, 1185.5 ± 47.3 ms, *p* < 0.0001; at 9–10 min, 1064.2 ± 80.7 ms, *p* < 0.05; Supplementary Fig. 9bii).

Lastly, the apnoea-duration index (ADI) of PNMT-AMPK-α1/α2 knockouts (1.5 ± 0.2) was significantly smaller compared to AMPK-α1/α2 floxed mice (2.9 ± 0.2, *p* < 0.05), but comparable to PNMT-Cre mice (2.3 ± 0.3, NS) during the first 5 min of 10-min exposures to severe hypoxia (Fig. 6c), although minute-by-minute analysis revealed significant attenuation of the ADI during the second (1.4 ± 0.3 compared to 2.8 ± 0.4 for PNMT-Cre, *p* < 0.0001) and third (1.9 ± 0.3 compared to 3.3 ± 0.6 for PNMT-Cre, *p* < 0.01) minutes of exposure to hypoxia (Supplementary Fig. 9ci). During the second half of 10-min exposures, however, the ADI was comparable for PNMT-AMPK-α1/α2 knockouts (1 ± 0.1, NS) and both sets of controls (PNMT Cre, 1.3 ± 0.2; AMPK-α1/α2 floxed, 1.5 ± 0.2), due in part to time-dependent and apnoea frequency–driven decreases in the ADI (Supplementary Fig. 9c). In contrast to PNMT-AMPK-α1/α2 knockouts and controls, during severe hypoxia, the ADI of TH-AMPK-α1/α2 knockouts was markedly augmented. When compared to AMPK-α1/α2 floxed mice, this was evident during the first (6.4 ± 0.4, *p* < 0.0001) and second (5.1 ± 0.5, *p* < 0.0001) 5-min periods of the 10-min exposures to severe hypoxia.

It would appear, therefore, that AMPK-dependent signalling pathways of noradrenergic networks facilitate the HVR and thus oppose ventilatory instability, hypoventilation, and apnoea during hypoxia, and in a manner that receives some form of negative feedback/input consequent to AMPK-dependent modulation of adrenergic networks.

## Discussion

Our previous investigations demonstrated that AMPK deletion in catecholaminergic cells attenuated the HVR and precipitated severe hypoventilation and apnoea, rather than hyperventilation [38]. By contrast, we have shown here that deletion of AMPK-α1/α2 in adrenergic cells moderately augmented the HVR, further stabilised respiratory rhythm, and reduced apnoea frequency during 10-min exposures to severe hypoxia. This indicates differential roles for AMPK-dependent modulation of adrenergic inputs and other catecholaminergic (i.e. dopaminergic and/or noradrenergic) inputs in regulating the HVR and thus whole-body oxygen and energy supply. It seems plausible, given reductions in apnoea frequency following AMPK deletion in adrenergic cells, that AMPK-dependent modulation of non-adrenergic catecholaminergic signalling pathways facilitates the HVR and protects against apnoea during hypoxia by enhancing ventilation, whereas AMPK-dependent modulation of adrenergic signalling pathways may, if anything, moderate the HVR in a manner that also contributes to the prolongation of apnoea, at least during the onset of hypoxia.

Neither AMPK-α1/α2 deletion in catecholaminergic nor adrenergic neurons markedly affected the augmenting phase of the HVR, which is consistent with the generally held view that this phase of the HVR is primarily mediated by carotid body afferent input responses [8, 34, 36]. Strikingly, however, the HVR of TH-AMPK-α1/α2 knockout mice was markedly attenuated throughout the sustained phase of exposures to severe poikilocapnic hypoxia, whereas the HVR of PNMT-AMPK-α1/α2 knockouts was moderately augmented. In each case, this was due to changes in breathing frequency, tidal volume, and thus minute ventilation relative to controls. When considered alongside our previous demonstration that AMPK deletion in catecholaminergic neurons attenuated the HVR where carotid body afferent inputs are normal, this finding adds further support to the view that direct modulation by hypoxia of brainstem respiratory networks aids coordination of the HVR in the longer term [7, 23, 28, 34, 36]. Nevertheless, that AMPK deletion in adrenergic neurons augmented the HVR was surprising given that the adrenergic ventrolateral C1 neurons have been proposed to form an integral part of the neural circuit that drives increases in breathing frequency during hypoxia in mice and rats [2, 5]. Considering this, our findings may point to the provision of an AMPK-dependent inhibitory input from the C2 and/or C3 neurons to the rCPGs, either directly or indirectly via moderation of afferent NTS inputs. Alternatively, AMPK may act to limit activity of C1 neurons during hypoxia, which is in line with the finding that AMPK slows action potential firing frequency in hippocampal neurons by direct phosphorylation and activation of K_V_2.1 [18].

Because AMPK deletion in *all* catecholaminergic cell types precipitated severe hypoventilation and thus revealed AMPK-dependent positive inputs, it therefore seems plausible that inhibitory adrenergic inputs may act to limit and/or periodically attenuate respiration triggered by hypoxic hyperventilation facilitated by AMPK-dependent regulation of noradrenergic and/or dopaminergic inputs, perhaps to limit respiratory alkalosis and aid efficient management of physiological CO_2_ levels [4, 12]. It is evident that attenuation and thus moderation of the HVR can be conferred through direct silencing of RTN neurons by respiratory alkalosis leading to consequent reductions in breathing frequency and tidal volume [4], although it should be noted that optogenetic inhibition of the RTN during hypoxia did not lead to further reductions in breathing frequency unless the hypoxic stimulus was supplemented with 3% CO_2_ or pharmacological agents that led to blood re-acidification [4]. Therefore, a contribution to those mechanisms that oppose respiratory alkalosis through AMPK-dependent regulation of adrenergic neurons is not beyond the bounds of possibility, given that the RTN can be inhibited through the release of inhibitory neuropeptides (such as enkephalin or neuropeptide Y) or signalling pathways involving GABAergic interneurons, that could be coordinated by the wider adrenergic network [26, 31].

Critically, AMPK deletion in adrenergic neurons also decreased the variability of inter-breath intervals during the first 3 min of the hypoxic exposures, and reduced apnoea frequency. Conversely one would expect that AMPK-dependent regulation of adrenergic pathways during hypoxia might ordinarily act to oppose the HVR and facilitate apnoea, which would lead to periodic cessations of ventilation that have been proposed to be the most rapid and efficient way to restore central CO_2_ levels during periods of intense respiratory effort [9]. Thus, during hypoxia, the balance of positive inspiratory drive from the carotid bodies and AMPK-dependent activation of brainstem hypoxia-responsive networks of noradrenergic/dopaminergic neurons on the one hand and AMPK-dependent activation of inhibitory inputs from adrenergic neurons on the other hand may coordinate HVR in a manner that aids maintenance of both oxygen supply and physiological CO_2_ levels.

In summary, AMPK facilitates the HVR through non-adrenergic catecholaminergic mechanisms. Whereas AMPK-dependent modulation of noradrenergic and/or dopaminergic networks may facilitate increases in ventilatory drive that shape the classical HVR, AMPK-dependent modulation of adrenergic networks may provide some form of negative feedback or inhibitory input to moderate HVR, which may protect against hyperventilation-induced hypocapnia and respiratory alkalosis [4, 12]. However, further studies will be required to determine whether this is indeed the case.

## Supplementary Information

Below is the link to the electronic supplementary material.

Supplementary file1 (PDF 4780 KB)

## Data Availability

All available data are included in this manuscript.

## References

[1] Abbott SB (2009). Photostimulation of retrotrapezoid nucleus phox2b-expressing neurons in vivo produces long-lasting activation of breathing in rats. J Neurosci : Off J Soc Neurosci.

[2] Abbott SB (2013). Selective optogenetic activation of rostral ventrolateral medullary catecholaminergic neurons produces cardiorespiratory stimulation in conscious mice. J Neurosci.

[3] Adachi T (2006). Mice with blunted hypoxic ventilatory response are susceptible to respiratory disturbance during hypoxia. Tohoku J Exp Med.

[4] Basting TM (2015). Hypoxia silences retrotrapezoid nucleus respiratory chemoreceptors via alkalosis. J Neurosci: Off J Soc Neurosci.

[5] Burke PG (2014). Optogenetic stimulation of adrenergic C1 neurons causes sleep state-dependent cardiorespiratory stimulation and arousal with sighs in rats. Am J Respir Crit Care Med.

[6] Burke PG, Kanbar R, Viar KE, Stornetta RL, Guyenet PG (2015). Selective optogenetic stimulation of the retrotrapezoid nucleus in sleeping rats activates breathing without changing blood pressure or causing arousal or sighs. J Appl Physiol.

[7] Curran AK (2000). Ventilatory responses to specific CNS hypoxia in sleeping dogs. J Appl Physiol.

[8] Day TA, Wilson RJ (2007). Brainstem PCO2 modulates phrenic responses to specific carotid body hypoxia in an in situ dual perfused rat preparation. J Physiol.

[9] Dempsey JA (2005). Crossing the apnoeic threshold: causes and consequences. Exp Physiol.

[10] Ebert SN (2004). Targeted insertion of the Cre-recombinase gene at the phenylethanolamine n-methyltransferase locus: a new model for studying the developmental distribution of adrenergic cells. Dev Dyn: an off Publ Am Assoc Anatomists.

[11] Evans AM, Mahmoud AD, Moral-Sanz J, Hartmann S (2016). The emerging role of AMPK in the regulation of breathing and oxygen supply. Biochem J.

[12] Foster GT, Vaziri ND, Sassoon CS (2001). Respiratory alkalosis. Respir Care.

[13] Gowans GJ, Hawley SA, Ross FA, Hardie DG (2013). AMP is a true physiological regulator of AMP-activated protein kinase by both allosteric activation and enhancing net phosphorylation. Cell Metab.

[14] Guyenet PG (2000). Neural structures that mediate sympathoexcitation during hypoxia. Respir Physiol.

[15] Guyenet PG (2013). C1 neurons: the body's EMTs. Am J Physiol Regul Integr Comp Physiol.

[16] Guyenet PG (2013). C1 neurons: the body's EMTs. Am J Phys - Regul, Integr Comp Physiol.

[17] Guyenet PG (2014). Regulation of breathing and autonomic outflows by chemoreceptors. Compr Physiol.

[18] Ikematsu N (2011). Phosphorylation of the voltage-gated potassium channel Kv2.1 by AMP-activated protein kinase regulates membrane excitability. Proc Natl Acad Sci U S A.

[19] Ivy CM, Scott GR (2017). Ventilatory acclimatization to hypoxia in mice: methodological considerations. Respir Physiol Neurobiol.

[20] Kalia M, Woodward DJ, Smith WK, Fuxe K (1985). Rat medulla oblongata. IV. Topographical distribution of catecholaminergic neurons with quantitative three-dimensional computer reconstruction. J Comp Neurol.

[21] Lantier L (2014). AMPK controls exercise endurance, mitochondrial oxidative capacity, and skeletal muscle integrity. FASEB J.

[22] Lindeberg J (2004). Transgenic expression of Cre recombinase from the tyrosine hydroxylase locus. Genesis.

[23] Mahmoud AD (2016). AMPK deficiency blocks the hypoxic ventilatory response and thus precipitates hypoventilation and apnea. Am J Respir Crit Care Med.

[24] Minson J, Llewellyn-Smith I, Neville A, Somogyi P, Chalmers J (1990). Quantitative analysis of spinally projecting adrenaline-synthesising neurons of C1, C2 and C3 groups in rat medulla oblongata. J Auton Nerv Syst.

[25] Nurse CA (2014). Synaptic and paracrine mechanisms at carotid body arterial chemoreceptors. J Physiol.

[26] Pilowsky PM (2008). Metabotropic neurotransmission and integration of sympathetic nerve activity by the rostral ventrolateral medulla in the rat. Clin Exp Pharmacol Physiol.

[27] Ross FA, MacKintosh C, Hardie DG (2016). AMP-activated protein kinase: a cellular energy sensor that comes in 12 flavours. FEBS J.

[28] Smith CA, Engwall MJ, Dempsey JA, Bisgard GE (1993). Effects of specific carotid body and brain hypoxia on respiratory muscle control in the awake goat. J Physiol.

[29] Smith JC, Abdala AP, Borgmann A, Rybak IA, Paton JF (2013). Brainstem respiratory networks: building blocks and microcircuits. Trends Neurosci.

[30] Souza G, Stornetta RL, Stornetta DS, Abbott SBG, Guyenet PG (2019). Contribution of the retrotrapezoid nucleus and carotid bodies to hypercapnia- and hypoxia-induced arousal from sleep. J Neurosci.

[31] Stornetta RL, Akey PJ, Guyenet PG (1999). Location and electrophysiological characterization of rostral medullary adrenergic neurons that contain neuropeptide Y mRNA in rat medulla. J Comp Neurol.

[32] Tankersley CG, Fitzgerald RS, Kleeberger SR (1994). Differential control of ventilation among inbred strains of mice. Am J Physiol.

[33] Tankersley CG, Elston RC, Schnell AH (2000). Genetic determinants of acute hypoxic ventilation: patterns of inheritance in mice. J Appl Physiol.

[34] Teppema LJ, Dahan A (2010). The ventilatory response to hypoxia in mammals: mechanisms, measurement, and analysis. Physiol Rev.

[35] Ward NL (2007). Cerebral angiogenic factors, angiogenesis, and physiological response to chronic hypoxia differ among four commonly used mouse strains. J Appl Physiol.

[36] Wilson RJ, Teppema LJ (2016). Integration of central and peripheral respiratory chemoreflexes. Compr Physiol.

[37] Evans AM, Hardie DG (2020) AMPK and the Need to Breathe and Feed: What's the Matter with Oxygen? Int J Mol Sci 21(10):3518. 10.3390/ijms21103518 10.3390/ijms21103518 PMC7279029 32429235

[38] Mahmoud AD (2014) Thesis: The loss of LKB1 and the AMP-activated protein kinase in catecholaminergic cells and the effect on the ventilatory response to hypoxia and hypercapnia. (University of Edinburgh, University of Edinburgh) 1–273.

[39] Franklin KBJ, Paxinos G (2008) *The mouse brain in stereotaxic coordinates* (Elsevier, Amsterdam ; London, ed. 3rd, 2008), pp. xxviii, 325

