# Supplementary material for: AMPK facilitates the hypoxic ventilatory response through non-adrenergic mechanisms at the brainstem
Source: Pflugers Arch. 2022 Jun 10;475(1):89–99. doi: 10.1007/s00424-022-02713-8 (PMC9816276; doi:10.1007/s00424-022-02713-8)
Supplement: Supplementary file 1 — Supplementary file1 (PDF 4780 KB) [file 424_2022_2713_MOESM1_ESM.pdf]

Supplementary Information

AMPK facilitates the hypoxic ventilatory response through non-adrenergic mechanisms at the brainstem

Sandy MacMillan[1], A. Mark Evans[1*]

[1]Centre for Discovery Brain Sciences, Hugh Robson Building, University of Edinburgh, Edinburgh, EH8 9XD, UK.

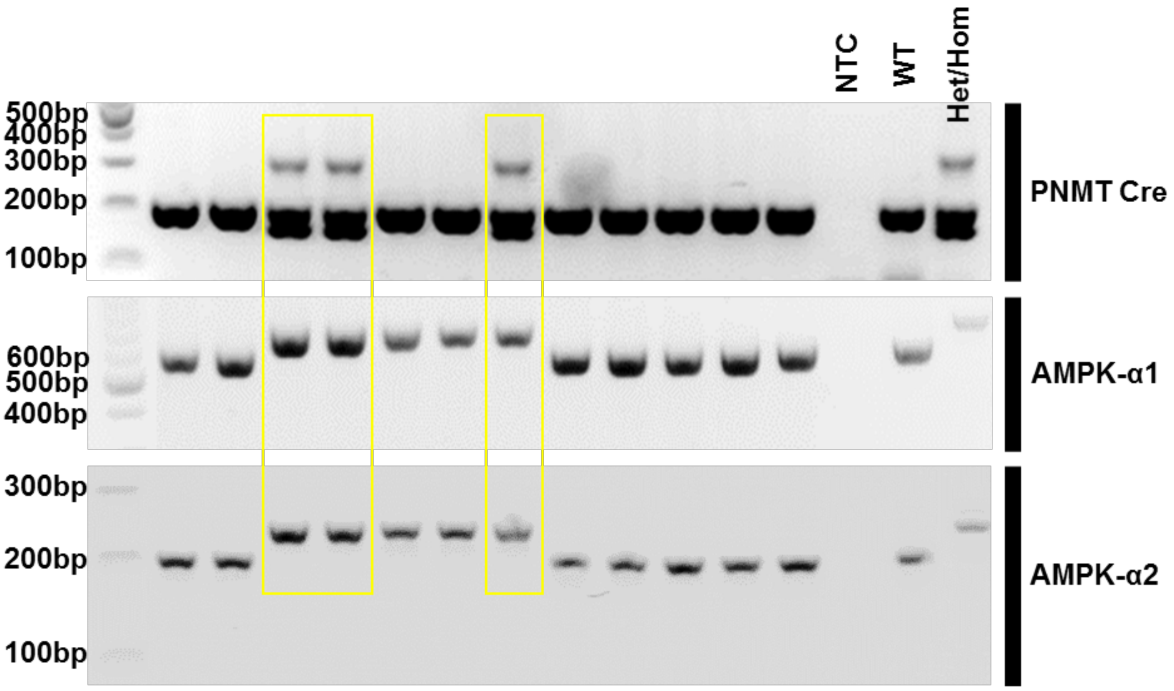

Supplementary Fig. 1 Genotype analysis confirms the expression of PNMT Cre and AMPK-α1$^{fl/fl}$ and AMPK-α2$^{fl/fl}$ catalytic subunits.

The products of targeted gene amplification obtained by polymerase chain reaction were run on a 2% agarose gel and visualised. The first lane was loaded with a 1kb DNA ladder, followed by 12 ear clip samples obtained from PNMT AMPK-α1/α2 knockout pups. NTC contains a no-template control, WT an ear clip sample from a wild-type C57Bl6 mouse, and Het/Hom was loaded with a samples from a mouse with heterozygous genetic modification of the PNMT gene and a homozygous genetic modification of the AMPK-α1 and -α2 subunits. The yellow boxes highlight three mice that were confirmed as PNMT Cre-driven AMPK-α1/α2 double knockouts.

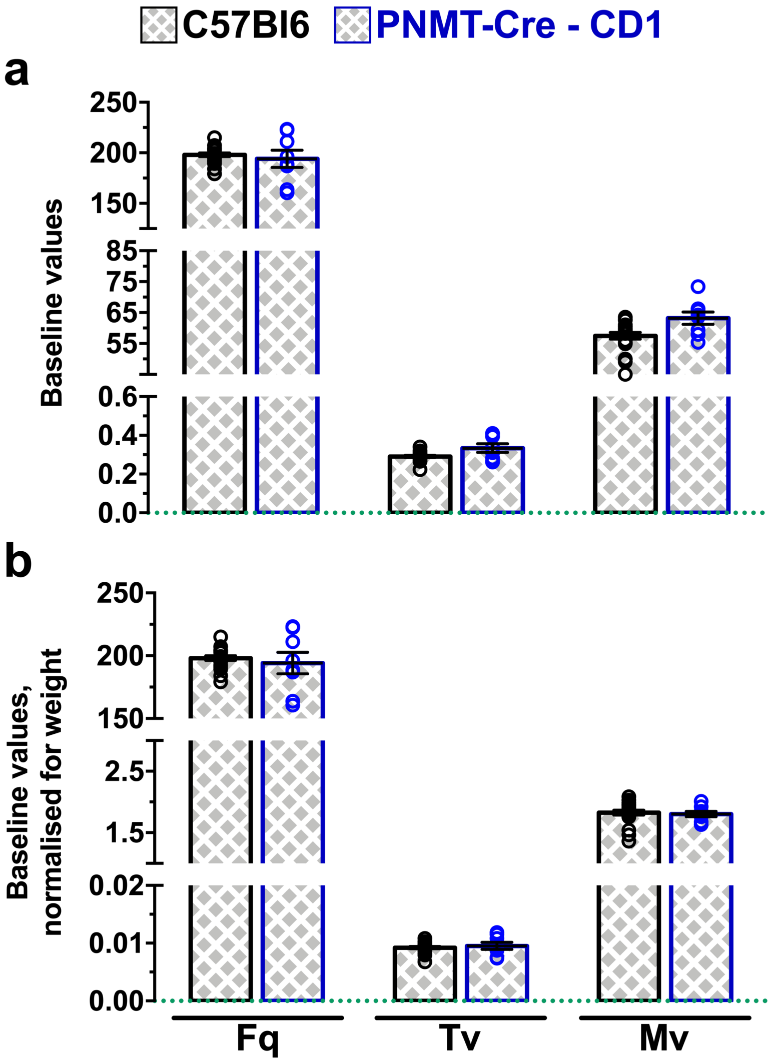

Supplementary Fig. 2 Pre-hypoxic baselines are comparable for C57Bl6 and PNMT-Cre – CD1 background controls

Bar charts show mean±SEM for (A) ‘raw’ baselines and (B) baselines normalised for weight of breathing frequency (Fq; breaths min^{-1}), tidal volume (Tv; ml) and minute ventilation (Mv; ml min^{-1}) that were selected prior to exposures to severe hypoxia (8% O_2) for wild-type C57Bl6 mice (black checked, n = 20 exposures from 5 mice), and CD1 mice expressing Cre under the Phenylethanolamine N-methyltransferase promoter (PNMT-Cre – CD1, blue checked, n = 8 exposures from 2 mice).

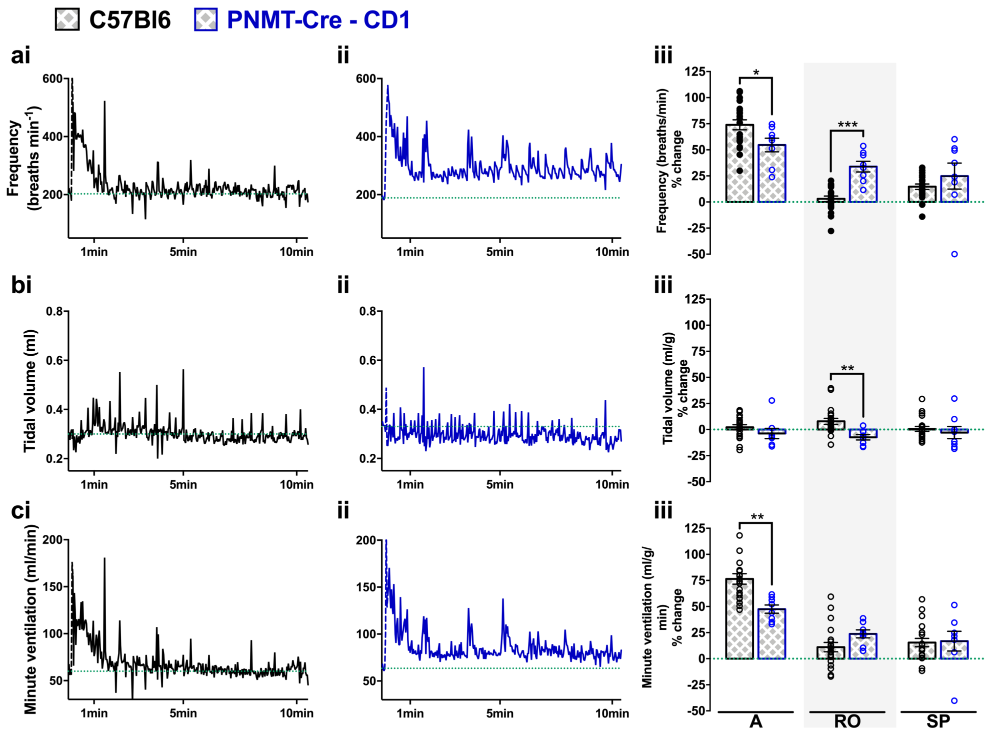

Supplementary Fig. 3: The hypoxic ventilatory response to severe hypoxia is background strain-specific.

Left and middle panels show raw example records of (A) breathing frequency (breaths min^{-1}), (B) tidal volume (ml) and (C) minute ventilation (ml/min) during 10min exposures to severe hypoxia (8% O_2) from (i) a wild-type C57Bl6 mouse and (ii) a CD1 mouse expressing Cre under the Phenylethanolamine N-methyltransferase reporter (PNMT-Cre – CD1) with a 2s sampling frequency. Dotted green lines indicate pre-hypoxic baseline frequencies, dashed lines the artefacts induced by gas exchange. Right panels (iii) show means±SEM for percentage changes during the peak of the augmenting phase (A, approximately 30s), the nadir of the Roll-Off (RO, approximately 100s), and the plateau of the sustained phase (SP, at 300s) of the hypoxic ventilatory response in C57Bl6 mice (black checked, n = 20 exposures from 5 mice) and PNMT-Cre – CD1 mice (blue checked, n = 8 exposures from 2 mice). *=p<0.05, **=p<0.01, ***=p<0.001.

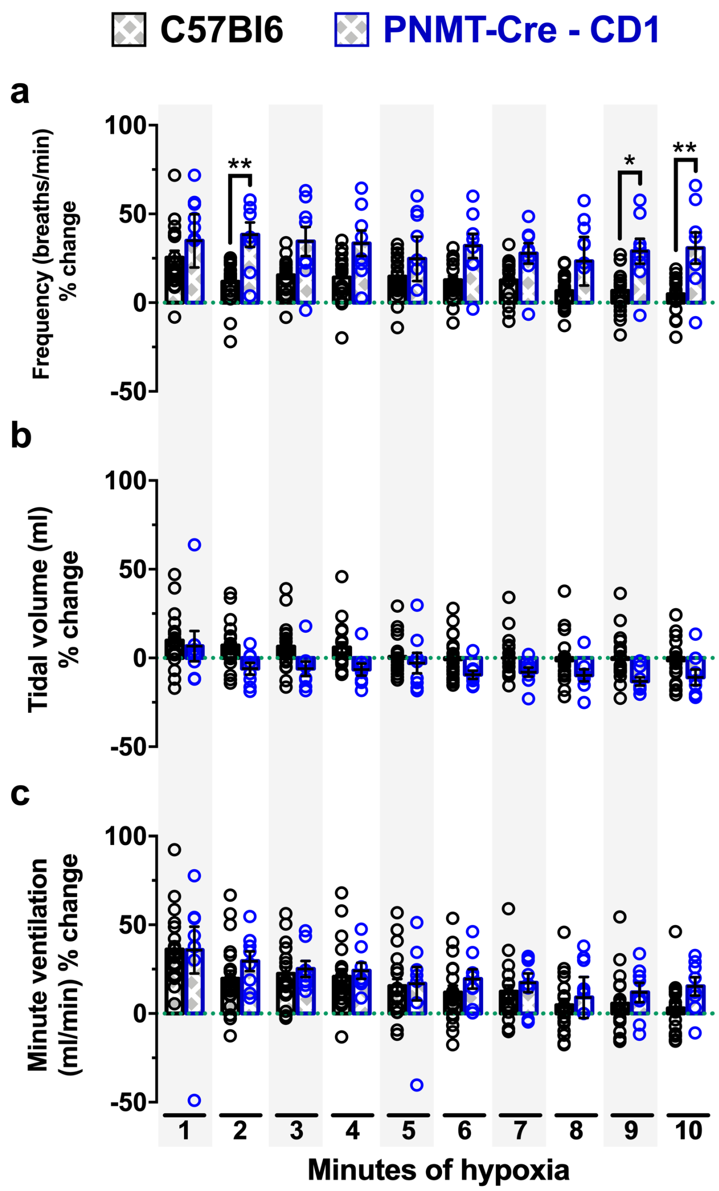

Supplementary Fig. 4: Minute-to-minute analysis of the hypoxic ventilatory response to severe hypoxia is background strain-specific.

Means±SEM for percentage change relative to normoxia (green dotted line) of (A) breathing frequency, (B) tidal volume, and (C) minute ventilation during 10min exposures to severe hypoxia (8% O_2) in wild-type C57Bl6 mice (black checked, n = 20 exposures from 5 mice) and CD1 mice expressing Cre under the Phenylethanolamine N-methyltransferase reporter (PNMT-Cre – CD1, blue checked, n = 8 exposures from 2 mice) measured at each full minute of hypoxia. *=p<0.05, **=p<0.01.

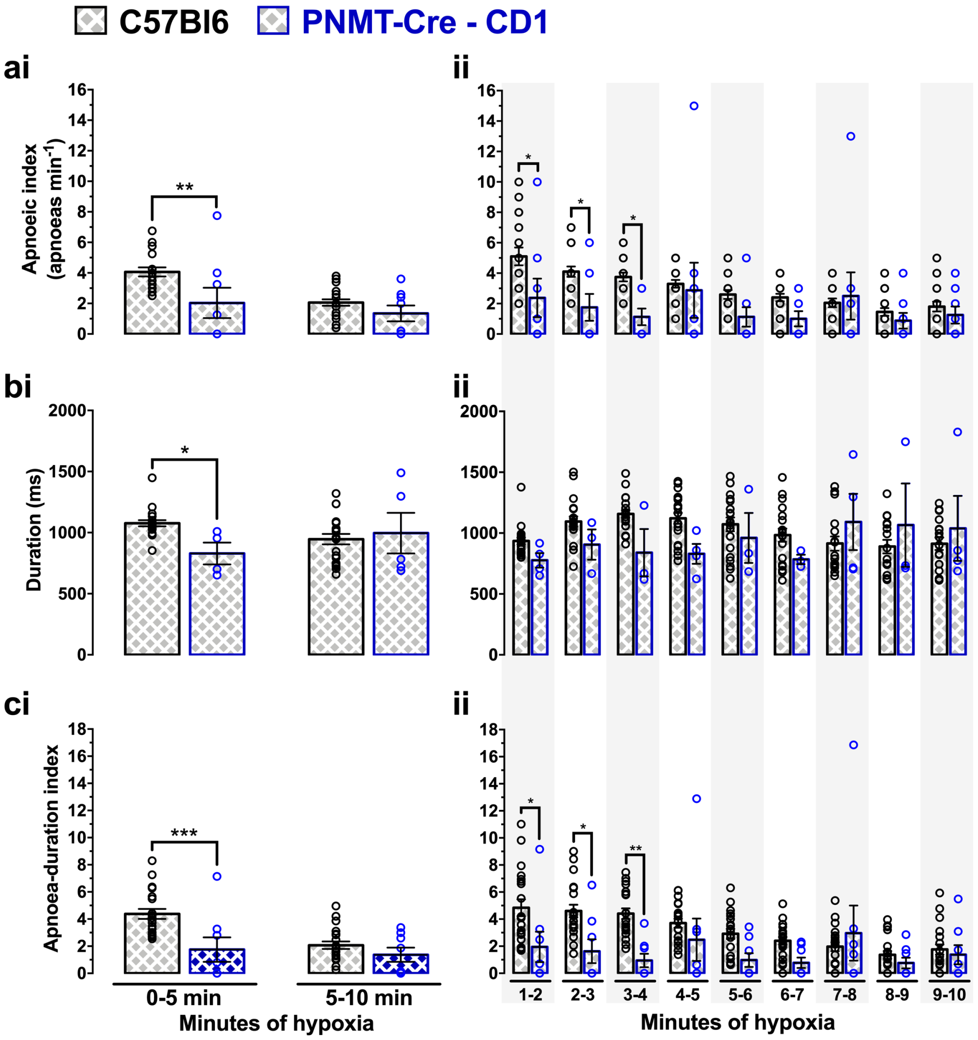

Supplementary Fig. 5: Hypoxia-evoked apnoea is background strain-specific.

Mean±SEM for the (A) apnoeic index (apnoeas min^{-1}), (B) apnoea duration (ms), and (C) apnoea-duration index during (i) the first and second half and (ii) for every 60s period of 10min exposures to severe hypoxia (8% O_2) in wild-type C57Bl6 mice (black checked, n = 20 exposures from 5 mice) and CD1 mice expressing Cre under the Phenylethanolamine N-methyltransferase reporter (PNMT-Cre – CD1, blue checked, n = 8 exposures from 2 mice). *=p<0.05, **=p<0.01, ***=p<0.001.

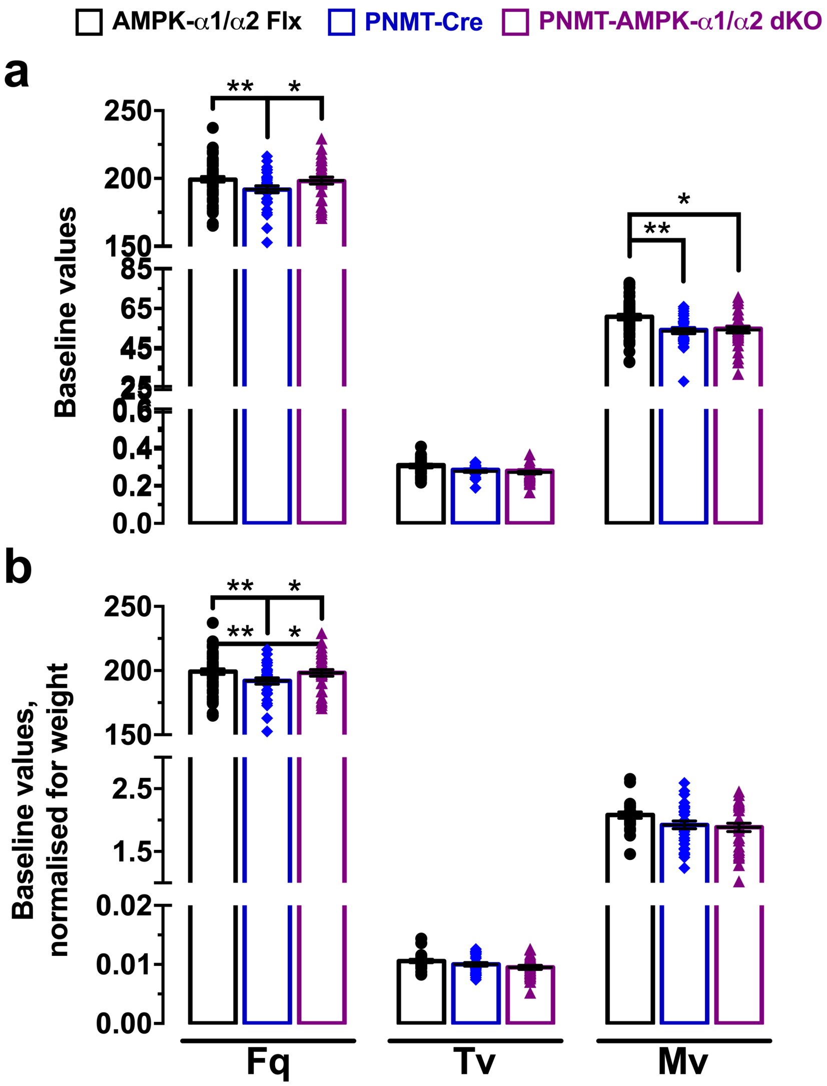

Supplementary Fig. 6: Differences in the pre-hypoxic baselines between the genotypes tested.

Bar charts show mean±SEM for (A) 'raw' baselines and (B) baselines normalised for weight of breathing frequency (Fq; breaths min^{-1}), tidal volume (Tv; ml) and minute ventilation (Mv; ml min^{-1}) that were selected prior to exposures to severe hypoxia (8% O_2) for AMPK-α1/α2 floxed mice (black, n = 58 exposures from 21 mice), mice expressing Cre under the Phenylethanolamine N-methyltransferase reporter (PNMT-Cre, blue, n = 32 exposures from 8 mice) and PNMT Cre driven AMPK-α1/α2 double knockout mice (PNMT-AMPK-α1/α2 knockouts, purple, n = 32 exposures from 8 mice). *=p<0.05, **=p<0.01.

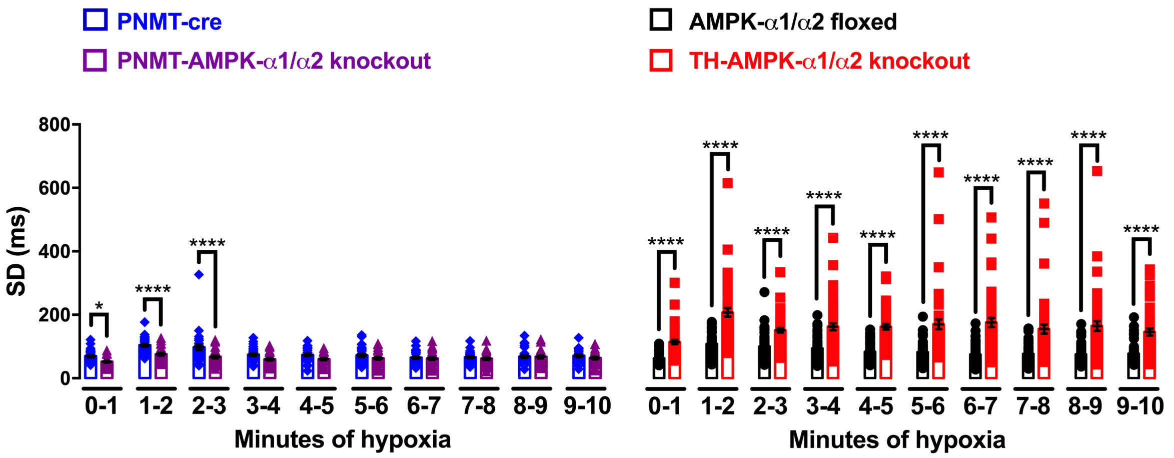

Supplementary Fig. 7: AMPK deletion in adrenergic cells reduces and AMPK deletion in catecholaminergic cells augments variability in inter-breath intervals during exposures to severe hypoxia

Bar charts show mean±SEM for the standard deviation (SD) of inter-breath intervals in mice expressing Cre under the Phenylethanolamine N-methyltransferase reporter (PNMT-Cre, blue, n = 32 exposures from 8 mice), PNMT Cre driven AMPK-α1/α2 double knockout mice (PNMT-AMPK-α1/α2 knockout, purple, n = 32 exposures from 8 mice), AMPK-α1/α2 floxed mice (black, n = 58 exposures from 21 mice), and TH Cre driven AMPK-α1/α2 double knockout mice (TH-AMPK-α1/α2 knockout, red, n = 49 exposures from 21 mice) for every 60s of 10min exposures to severe hypoxia (8% O_2). *=$p<0.05$, ****=$p<0.0001$.

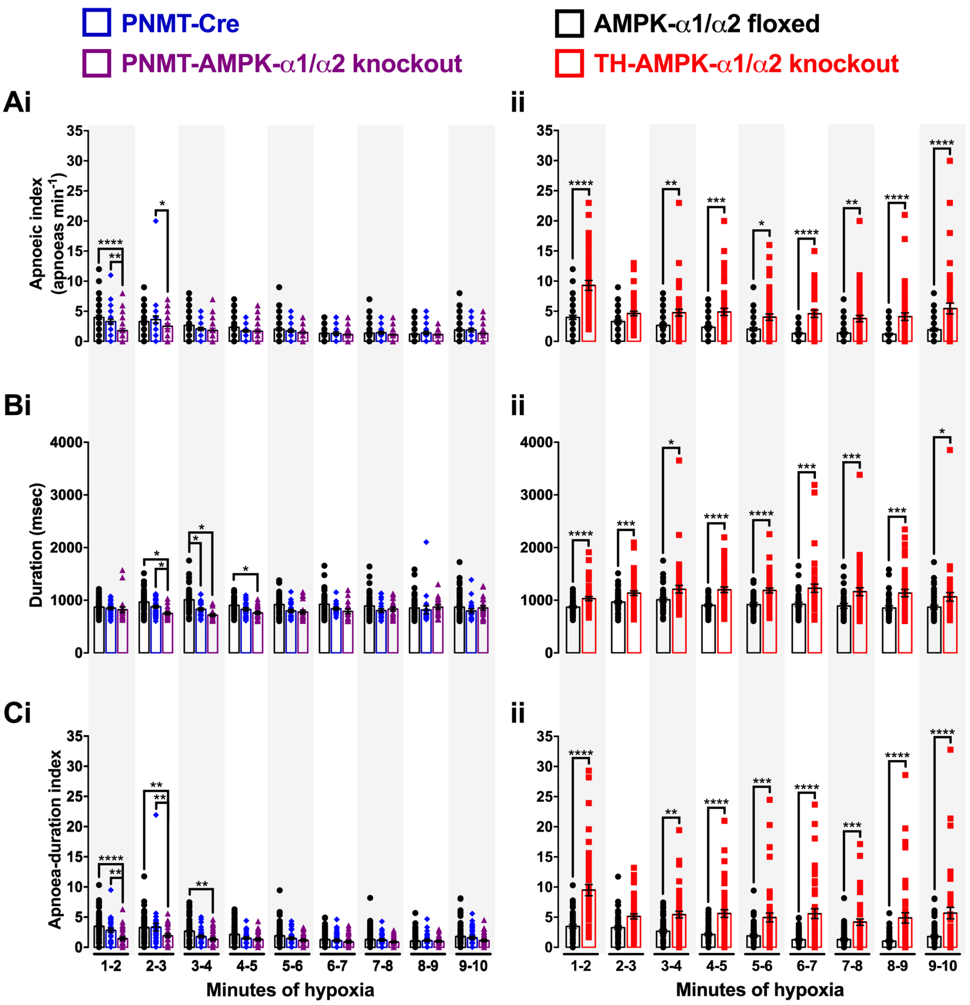

Supplementary Fig. 8: Minute-by-minute analyses of apnoeas in response to severe hypoxia across all genotypes tested.

Mean±SEM for (A) apnoeic index (min^{-1}), (B) apnoea duration (msec) and (C) apnoea-duration index (frequency x duration) measured every 60s of 10min exposures to severe hypoxia (8% O_2) for PNMT-Cre mice (blue, n = 32 exposures from 8 mice), PNMT Cre driven AMPK-α1/α2 double knockout mice (PNMT-AMPK-α1/α2 dKO, purple, n = 32 exposures from 8 mice), AMPK-α1/α2 floxed mice (black, n = 58 exposures from 21 mice), and TH Cre driven AMPK-α1/α2 double knockout mice (TH-AMPK-α1/α2 knockout, red, n = 49 exposures from 21 mice). *=p<0.05, **=p<0.01, ***=p<0.001, ****=p< 0.000
